# Case Report: Successful Treatment of Alopecia Universalis With Tofacitinib and Increased Cytokine Levels: Normal Therapeutic Reaction or Danger Signal?

**DOI:** 10.3389/fimmu.2022.904156

**Published:** 2022-06-20

**Authors:** Ling Yu, Huiqian Yu, Shuai Zhang, Yanzhao Hao, Shoumin Zhang

**Affiliations:** Department of Dermatology, Henan Provincial People’s Hospital, Zhengzhou University People’s Hospital, Henan University People’s Hospital, Zhengzhou, China

**Keywords:** alopecia universalis, tofacitinib, hair loss, Janus kinase inhibitor, cytokines

## Abstract

Alopecia universalis (AU) is an autoimmune disorder characterized by non-scarring hair loss in the scalp, eyebrows, beard, and nearly the entire body, negatively affecting patient prognosis. Available treatments are usually unsatisfactory. The autoimmune attacks of hair follicles induced by CD8+ T cells and the collapse of hair follicle immune privilege are believed to be the leading causes of AU. Additionally, interferon (IFN)-γ plays an important role in triggering the collapse of hair follicle immune privilege and impairing hair follicle stem cells. Furthermore, the upregulation of Janus kinase (JAK)3 and phospho-signal transducer and activator of transcription (pSTAT)3/STAT1 in alopecia areata patients suggest that JAK inhibitors can be a potentially promising choice for AU patients for the reason that JAK inhibitors can interfere with JAK-STAT signaling pathways and inhibit IFN-γ. Herein, we report a case of AU successfully treated with tofacitinib. However, this beneficial response in the patient was accompanied by a remarkable increase in peripheral blood cytokine levels during tofacitinib treatment.

## Introduction

Alopecia universalis (AU) is an autoimmune disorder characterized by non-scarring hair loss in the scalp, eyebrows, and beard, potentially resulting in complete hair loss all over the body. AU’s treatment options are usually unsatisfactory, and no specific effective therapies are available (1). The exact etiopathogenesis is unclear, but the collapse of the immune system and autoimmune attack of hair follicles induced by CD8+ T cells are believed to be the leading causes of AU (2). Gene expression of inflammatory markers (interleukin [IL]-2, Janus kinase [JAK]3, and IL-15), T helper type (Th) 1 pathway cytokines (interferon [IFN]-γ), and Th2 pathway cytokines (IL-13) increase in the lesional scalp of alopecia areata (AA) patients. Meanwhile, phospho-signal transducer and activator of transcription (pSTAT)3/STAT1 is also upregulated (3, 4). The serum cytokine level of IFN-γ is also significantly increased (5), suggesting that JAK inhibitors can be a potentially beneficial choice for AU patients due to JAK inhibitors interfering with the JAK-STAT signaling pathway and inhibiting IFN-γ. Herein, we report a case of AU successfully treated with tofacitinib. However, a remarkable increase in peripheral blood cytokine levels following tofacitinib treatment in the AU patient was observed.

## Case Description

**Figure 1:**
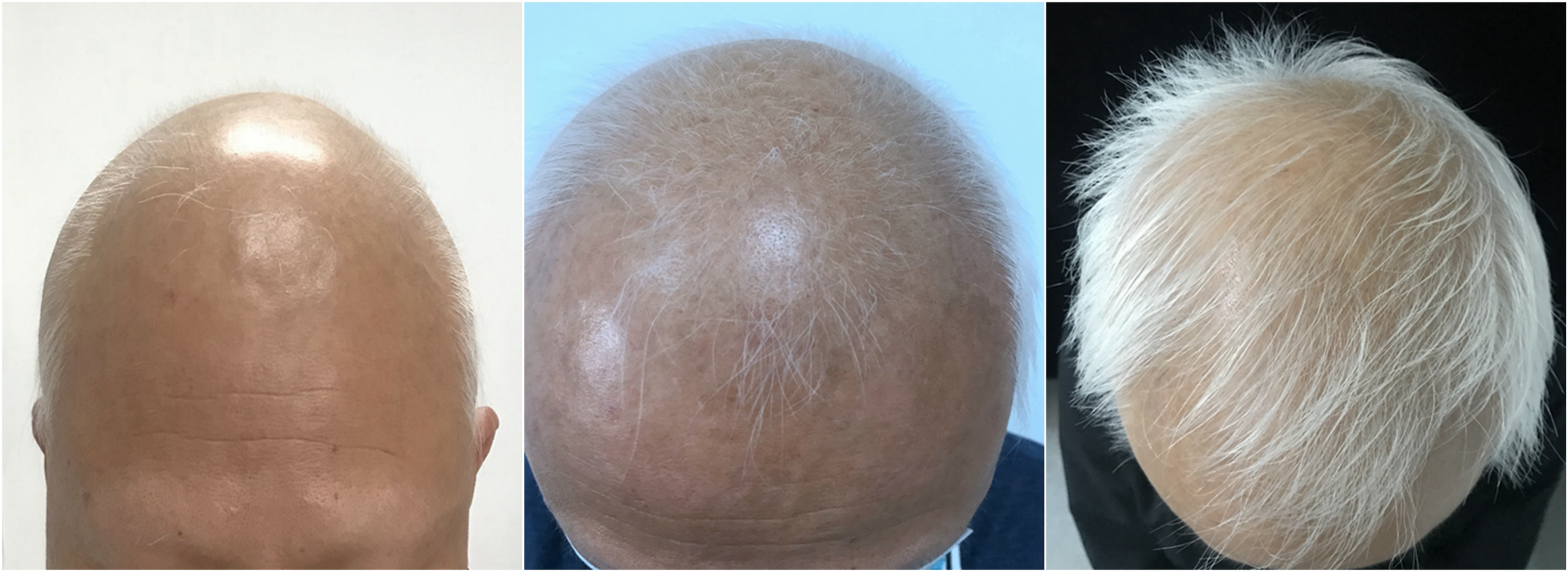
The clinical manifestation of the patient’s hair after tofacitinib treatment; at 8, 16, and 24 weeks, the hair has regrown gradually.

**Figure 2:**
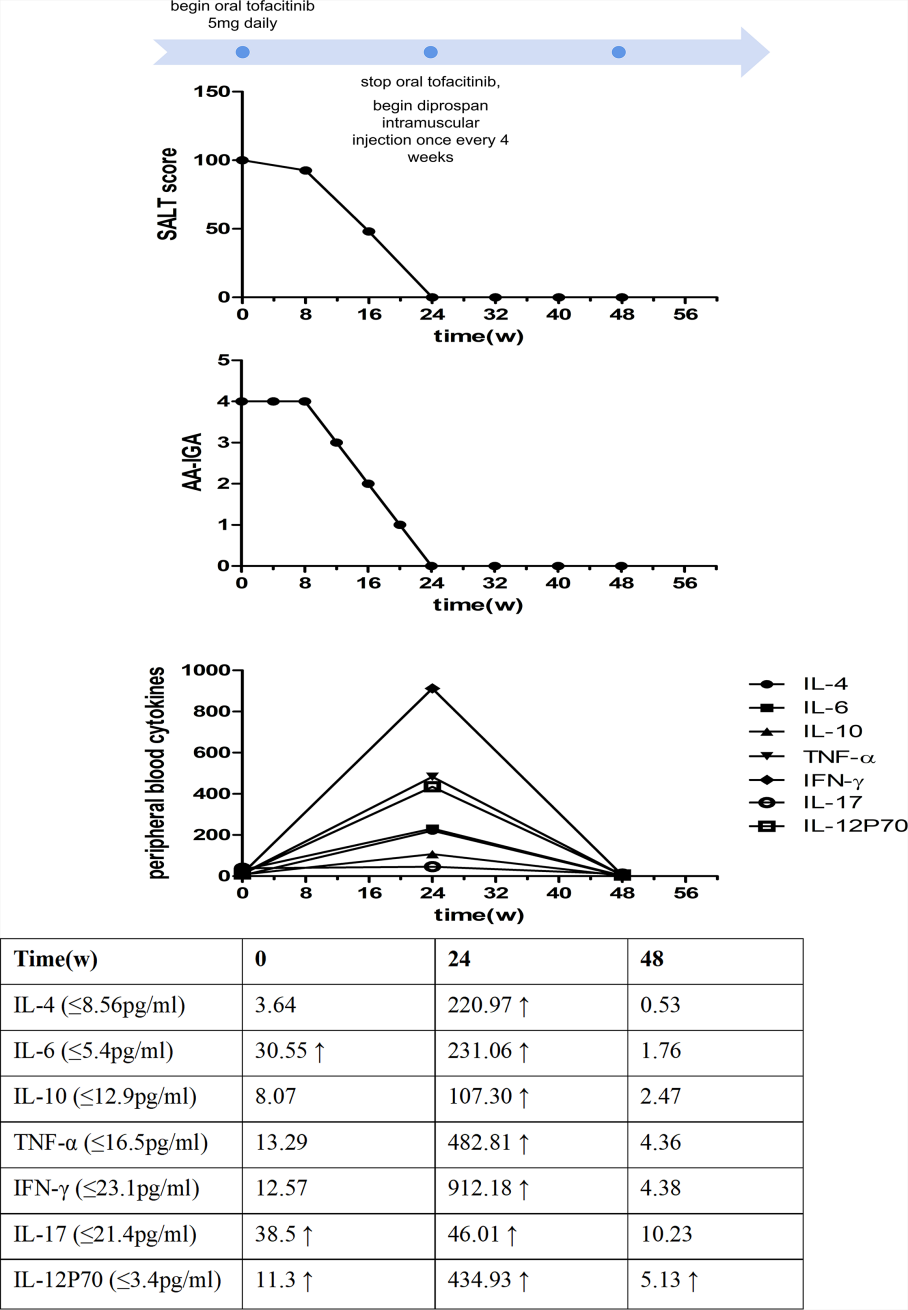
Time course of the treatment and changes in the Severity of Alopecia Tool (SALT) score, the Alopecia Areata Investigator Global Assessment (AA-IGA™), and the levels of peripheral blood cytokines.

A 53-year-old man visited our clinic complaining of quickly worsening hair loss in the scalp, eyebrows, beard, armpit hair, groin, and nearly his entire body 5 months ago, seriously affecting his appearance and increasing his psychological burden. Other than these outcomes, he was healthy without any medical history or family history of similar diseases except for transient urticaria 3 months ago. No previous treatments were administered to the patient. Skin examination showed hair loss in the scalp, eyebrows, beard, and the rest of his body (**Figure 1**). The Severity of Alopecia Tool (SALT) score (6) and the Alopecia Areata Investigator Global Assessment (AA-IGA™) score (7) was used to assess his hair loss. His SALT score was 100, and AA-IGA™ score was 4, which suggested that his condition was severe. Routine analysis of hemogram, hepatic and renal function, corticosteroid hormone, serum IgE, and T-SPOT.TB tests (T-SPOT) were all normal. We also examined his peripheral blood T-cell subsets, which showed that CD3+ T cells, CD3+CD4+ T cells, and CD4+/CD8+ T cells were normal except for CD3+CD8+ T cells, which were slightly increased. Simultaneously, peripheral blood cytokines were examined using a commercial multiple cytokine detection kit [multiple microsphere flow cytometry] (qdraisecare, China) to examine the cytokine level. The normal values of the cytokine level were defined based on the serum cytokine level of 198 healthy people. Results showed that IL-6, IL-17, and IL12p70 increased, while IL-4, IL-10, IFN-γ, and tumor necrosis factor-α (TNF-α) levels were within the normal range (**Figure 2**). According to the typical clinical manifestation and medical history, the patient was diagnosed with AU.

After written informed consent was obtained, the patient was administered 5 mg of oral tofacitinib daily. Fortunately, a rapid and significant therapeutic effect was observed after the treatment, and his hair regrew gradually, although the color of the hair was white. No systemic side effects were found after treatment except slightly elevated glutamic-pyruvic transaminase (ALT) levels and blood lipid levels. Twenty-four weeks after tofacitinib treatment, the SALT and AA-IGA™ scores decreased significantly to zero (**Figure 2**). We reexamined his peripheral blood T-cell subsets and found these values returned to normal. However, his peripheral blood cytokines were significantly increased, including Th1 cytokines IFN-γ, TNF-α, IL12p70, Th2 cytokines IL-4, IL-6, IL-10, and Th17 cytokines IL-17. IFN-γ was found to have increased 72 times compared with the baseline before tofacitinib treatment (**Figure 2**). Considering the patient’s safety and benefits observed with the patient’s regrown hair, oral tofacitinib was discontinued, and Diprospan (7 mg/ml, 1 ml, contains betamethasone disodium phosphate 2 mg and betamethasone dipropionate 5 mg) intramuscular injections were administered once every 4 weeks to sustain the effect. Twenty-four weeks after Diprospan treatment, his hair growth was maintained. We reexamined his peripheral blood cytokines and found that all the cytokines decreased to normal, with only IL12p70 remaining slightly above the standard level. However, this value still decreased compared to the baseline (**Figure 2**). The patient was very satisfied with both tofacitinib and Diprospan treatment, and the frequency of the Diprospan treatment was decreased gradually. No adverse effects were reported during the treatment or the follow-up.

## Discussion

AU is the most severe autoimmune disease affecting hair follicles of the scalp and the whole body and is mediated by CD8+ T cells (8). However, its exact pathogenesis remains unclear. Complex immunology, genetics, epigenetics, various environmental factors, and oxidative stress are suggested to participate in the development of the disease (9), especially as it relates to the collapse of hair follicle immune privilege, which is believed to be the leading cause (2). Current traditional treatments, including corticosteroids, immunosuppressive agents, topical minoxidil, and contact immunotherapy, show limited effects with adverse reactions. Recent studies have suggested that JAK inhibitors could be a promising treatment option for inflammatory diseases (1). Specifically, they can interfere with T cell-mediated inflammatory signaling pathways to mediate these outcomes. Tofacitinib, a JAK inhibitor, mainly acts on JAK 1 and 3 and has been previously investigated in AA treatment. These studies show that it is well-tolerated and effective for severe and recalcitrant cases without any reported serious adverse effects, although tofacitinib is unable to maintain a durable response when treatment is stopped (10). After tofacitinib treatment, our patient’s hair regrew gradually, and the SALT and AA-IGA™ scores decreased significantly (**Figure 2**). The beneficial response to regrowth of our patient’s hair suggests that tofacitinib can be an optimal choice for AU.

Notably, the significant increase in cytokine levels in peripheral blood may be of concern. Due to the potential of inducing other unknown adverse reactions, we discontinued tofacitinib following the treatment period. Cytokine levels decreased after discontinuation, suggesting that tofacitinib increased cytokine levels. Studies have shown that Th1, Th2, and Th17 cells may contribute to AA development (11), which is consistent with our findings. Serum Th1 cytokines, including IFN-γ, TNF-α, and IL-12p70, and serum Th2 cytokines IL-4, IL-6, IL-10, and Th17 cytokines IL-17 participated in the progression of AU. Tofacitinib can inhibit cytokines in hair follicles of AA patients, including IL-2, IL-4, IL-7, IL-15, IL-21, and IFN-γ, by blocking the STAT phosphorylation and disrupting the signaling pathway of JAK 1/3 (10). However, studies on peripheral blood cytokine changes after tofacitinib treatment are still lacking. Only one study evaluated serum IL-2, IL-4, IL-15, and IL-17 in patients with AA, in which all were observed to decrease after tofacitinib treatment (12), which was not consistent with our results. To our knowledge, no studies on changes in cytokine levels before and after tofacitinib treatment in AU patients have been reported. Our case is the first one exploring the cytokine level change of tofacitinib treatment in a patient with AU. However, because this study involved only one patient, we are limited by the small sample value. Whether there are any different reactions to tofacitinib between AA and AU requires further clarification and larger sample populations to investigate its reliability.

Studies on mouse models demonstrated that IFN-γ, a JAK-STAT (signal transducer and activator)-dependent cytokine, was crucial for the development of AA. Intravenous injection of IFN-γ could induce AA-like hair loss in C3H/HeJ mice (13). As previously demonstrated, JAK inhibitors interfere with JAK-STAT signaling pathways and inhibit IFN-γ (8). Therefore, oral tofacitinib could prevent and treat AA *via* these mechanisms. However, our case’s peripheral blood IFN-γ level increased significantly during hair regrowth after tofacitinib treatment. Therefore, the exact pathogenesis of cytokine level increase and whether it is a normal therapeutic reaction or a potential safety concern have not been comprehensively elucidated and requires further investigation.

## Conclusion

Overall, tofacitinib has shown a remarkable effect in treating AU, which offers a potentially optimal choice for AU patients. However, we still need to evaluate its safety and determine why cytokine levels increase and whether it will adversely affect the patient. Further studies and more extensive population research are needed to support its long-term efficacy and safety for the potential treatment of AU.

## Data Availability Statement

The original contributions presented in the study are included in the article/supplementary material. Further inquiries can be directed to the corresponding author.

## Ethics Statement

The studies involving human participants were reviewed and approved by Henan Provincial People’s Hospital Medical Ethics Committee. The patients/participants provided their written informed consent to participate in this study. Written informed consent was obtained from the individual(s) for the publication of any potentially identifiable images or data included in this article.

## Author Contributions

LY and HY researched the data, contributed to the discussion, wrote the manuscript, and reviewed the manuscript. SZ and YH researched the data and contributed to the discussion. SMZ reviewed the manuscript. All authors contributed to the article and approved the submitted version.

## Conflict of Interest

The authors declare that the research was conducted in the absence of any commercial or financial relationships that could be construed as a potential conflict of interest.

## Publisher’s Note

All claims expressed in this article are solely those of the authors and do not necessarily represent those of their affiliated organizations, or those of the publisher, the editors and the reviewers. Any product that may be evaluated in this article, or claim that may be made by its manufacturer, is not guaranteed or endorsed by the publisher.
